# Artificial Intelligence in Veterinary Clinical Pathology—An Introduction and Review

**DOI:** 10.1111/vcp.70012

**Published:** 2025-06-03

**Authors:** Samuel V. Neal, Daniel G. Rudmann, Kara N. Corps

**Affiliations:** ^1^ Department of Veterinary Biosciences, College of Veterinary Medicine Ohio State University Columbus Ohio USA; ^2^ Moderna Inc Pathology Cambridge Massachusetts USA

**Keywords:** AI, cytology, deep learning, digital pathology, machine learning

## Abstract

Artificial intelligence (AI), particularly through machine learning and deep learning, presents opportunities for the enhancement of the workflow of the veterinary clinical pathologist. This review introduces basic concepts in AI in a nontechnical manner and explores the qualification and integration of AI in veterinary clinical pathology. The veterinary clinical pathologist must play an active role in defining the intended use, design, and qualification of these methods as well as the plan for monitoring their responsible application in practice.

**Abbreviations**
AGI: artificial general intelligence
AI: artificial intelligence
ANI: artificial narrow intelligence
ANN: artificial neural network
ASI: artificial super intelligence
AUC: area under the curve
BAL: bronchoalveolar lavage
CFR: code of federal regulations
CKD: chronic kidney disease
CLIA: Clinical Laboratory Improvement Amendments
CNN: convolutional neural network
DL: deep learning
DNN: deep neural network
EHR: electronic health record
FDA: Food and Drug Administration
FIP: feline infectious peritonitis
GCP: good clinical practices
GLP: good laboratory practices
GRP: good research practices
LLM: large language model
MAE: mean absolute error
ML: machine learning
MSE: mean squared error
PCA: principal component analysis
POC: point‐of‐care
RAG: retrieval‐augmented generation
RMSE: root mean‐squared error
RNN: recurrent neural network
ROC: receiver operating characteristic
ViT: vision transformer
WSI: whole slide image
XAI: explainable artificial intelligence

## 1 Introduction

The proliferation of artificial intelligence (AI) can seem overwhelming, especially for those navigating its complexities in medicine. However, a robust understanding of the core principles of AI is both achievable and generally sufficient for practical applications. For veterinary clinical pathologists, a solid grasp of AI fundamentals is not merely advantageous but requisite. Responsible development and utilization of high‐performance AI‐based solutions mandate active involvement from professionals like veterinary clinical pathologists with both domain expertise and a foundational understanding of AI principles. This review provides a nontechnical overview of the fundamental principles of AI and its existing and potential contributions to veterinary clinical pathology.

The complexity and staggering breadth of the field of AI continue to advance at a remarkable rate. Since the conception of AI in the 1950s, with John McCarthy coining the term AI to describe “the science and engineering of making intelligent machines,” AI has expanded from models built on simple if‐then rules to sophisticated systems that can learn from data, adapt to new situations, and perform tasks that, until recently, were thought to require human intelligence [1]. This expansion in capability has led to a recent surge in the development of AI applications, with nearly 500 000 AI publications in 2021 alone [2].

Although much of this growth has been driven by applications in other industries, the potential for AI to assist veterinary clinical pathologists has been clear for decades, evidenced by AI tools like PAPNET in the 1990s, which assisted human cytopathologists in screening cervical cytology slides for abnormalities [3]. This potential continues to expand as veterinary clinical pathology is digitalized and electronic health records (EHRs) and whole‐slide images (WSIs) proliferate, creating large, high‐quality data sets—the foundation for the development of AI‐based tools. Such tools have the potential to help veterinary clinical pathologists integrate and interpret complex data sets in new and possibly more efficient ways.

## 2 Defining AI

Despite the ubiquity of the term “Artificial Intelligence,” AI has no universally agreed upon definition [4, 5]. This largely results from a lack of consensus on the definition of intelligence itself, the multi‐disciplinary nature of AI, and the pace of advancements [5]. For the purpose of this review, AI is considered “a set of technologies that enable computers to perform a variety of advanced functions” that “mimic human‐like cognitive functions such as learning and problem‐solving” [6, 7].

While technological advancements, breakthroughs in AI algorithm architecture, and increased data availability have significantly expanded AI capabilities in recent years, it is widely accepted that all existing systems still fall under the umbrella of artificial narrow intelligence (narrow or weak AI; ANI). ANI is AI that can perform a specific task or set of similar tasks as well or better than a human [7, 8]. Artificial general intelligence (general or strong AI; AGI) and artificial super intelligence (ASI) are theoretical goals. AGI refers to systems that can generalize to perform any task that a human can [7, 8]. ASI refers to a system that cannot only generalize to any situation but can outperform human experts in any domain [7]. Table 1 contains practical definitions of AI terms used throughout this review.

**TABLE 1:** Practical definitions of commonly used terms in artificial intelligence.

Term	Definition
Artificial general intelligence (AGI)	Theoretical systems that can generalize to perform any task that a human can
Artificial intelligence (AI)	“A set of technologies that enable computers to perform a variety of advanced functions” [6]
Artificial narrow intelligence (ANI)	AI that can perform a specific task or set of similar tasks as well or better than a human
Artificial neural network (ANN)	ML model inspired by biological neural networks composed of interconnected nodes
Artificial super intelligence (ASI)	Theoretical systems that can generalize to any situation and outperform human experts in any domain
Balanced accuracy	The arithmetic mean of sensitivity and specificity in binary classifications and the average sensitivity across each class in multiclass classifications
Classification	A supervised learning approach where the model is trained to classify input data into predefined discrete categories
Clustering	An unsupervised learning technique used to group data points that are similar
Computer vision	Field centered around enabling computers to analyze and interpret visual data
Convolutional neural network (CNN)	DNN specifically designed to handle grid‐like data such as images using convolutional layers to extract features
Deep neural network (DNN)	ANN with multiple hidden layers between the input and output layer enabling the modeling of complex data relationships
Dimensionality reduction	An unsupervised learning technique used to reduce the number of input variables in a data set while retaining essential information
*F*_1_ Score	The harmonic mean of precision and sensitivity, taking into account both false positives and negatives
Foundation model	A large‐scale, pretrained model that serves as a versatile base for a wide range of downstream tasks without needing task‐specific training from scratch
Hallucination	The phenomenon where a model generates output not grounded in reality or inconsistent with input data
Hyperparameters	Configuration settings used to tune and guide the learning process of a machine learning model, which are set before the training process begins and directly affect model performance and behavior
Imputation	The process of replacing missing data with substituted values
K‐fold cross‐validation	A method to accurately assess model performance where the training data set is split into k equal parts and the model is trained and tested k times, using each part as a test set once and as part of the training set k − 1 times
Large language model (LLM)	AI model designed to understand and generate human‐like text by leveraging transformer architectures
Machine learning (ML)	A subtype of AI where systems learn directly from data to perform tasks rather than needing to be explicitly programmed
Model drift	Degradation of model performance due to changes in the data
Multimodal	The integration or combination of multiple modes of data
Overfitting	When a model learns training data too well leading to poor performance on unseen data
Oversampling	The act of increasing the number of instances in a minority class to counteract imbalanced data
Principal component analysis (PCA)	A common method of dimensionality reduction where a larger set of correlated variables is reduced in number while retaining variables that still collectively explain most of the variability in the data set
Recurrent neural network (RNN)	DNN designed to handle sequential data such as time series predictions by maintaining an internal memory state
Regression	A supervised learning approach where the model is trained to predict continuous outcomes based on input data
Reinforcement learning	A subset of ML where an agent learns to make decisions by taking actions in an environment to achieve some goal
Retrieval‐augmented generation (RAG)	Optimization of an AI model through incorporation of a retrieval system and a curated database allowing for models to exhibit domain‐specific expertise
Self‐supervised learning	A subset of ML where models generate their own labels from the data itself
Semi‐supervised learning	A subset of ML where a small amount of labeled data and a large amount of unlabeled data are used for training
Supervised learning	A subset of ML where models are trained on labeled data
Transformer	DNN capable of capturing dependencies across entire sequences of data through self‐attention mechanisms
Underfitting	When a model is too simple to capture the complexity of data, leading to poor performance on both training and unseen data
Undersampling	The act of reducing the number of instances in a majority class to counteract imbalanced data
Unsupervised learning	A subset of ML where models are trained on unlabeled data
Vision transformer (ViT)	Transformer architecture designed for processing visual data

## 3 Machine Learning

**FIGURE 1:**
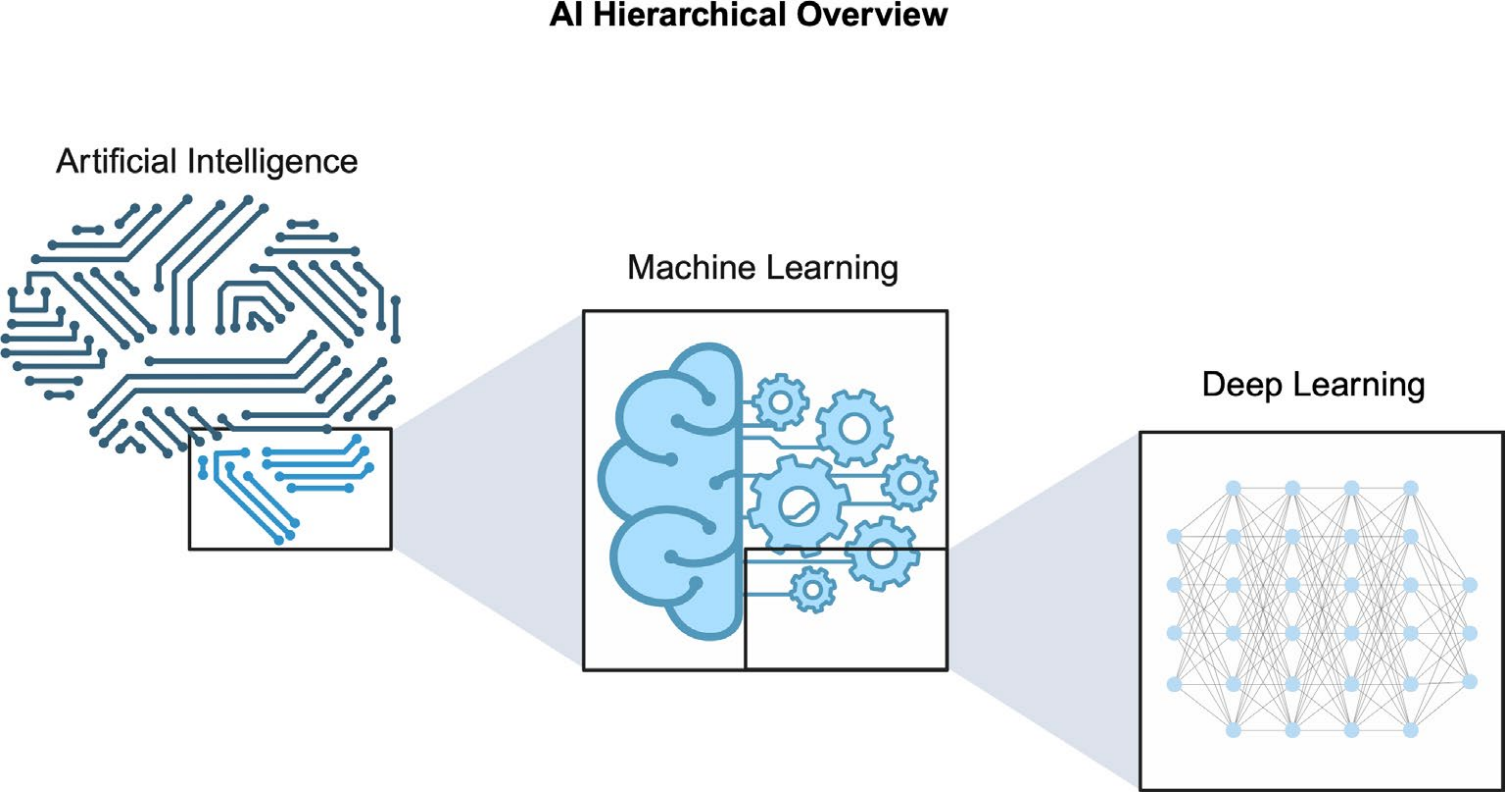
AI hierarchical overview. Artificial intelligence is a broad field encompassing many different computational methods to perform tasks historically thought to require human intelligence. Machine learning is a subset of artificial intelligence focused on algorithms that learn from data rather than being explicitly programmed. Deep learning is a subset of machine learning that involves machine learning algorithms that utilize the neural network architecture with multiple layers, termed deep neural networks (discussed later). Created with BioRender.com.

**FIGURE 2:**
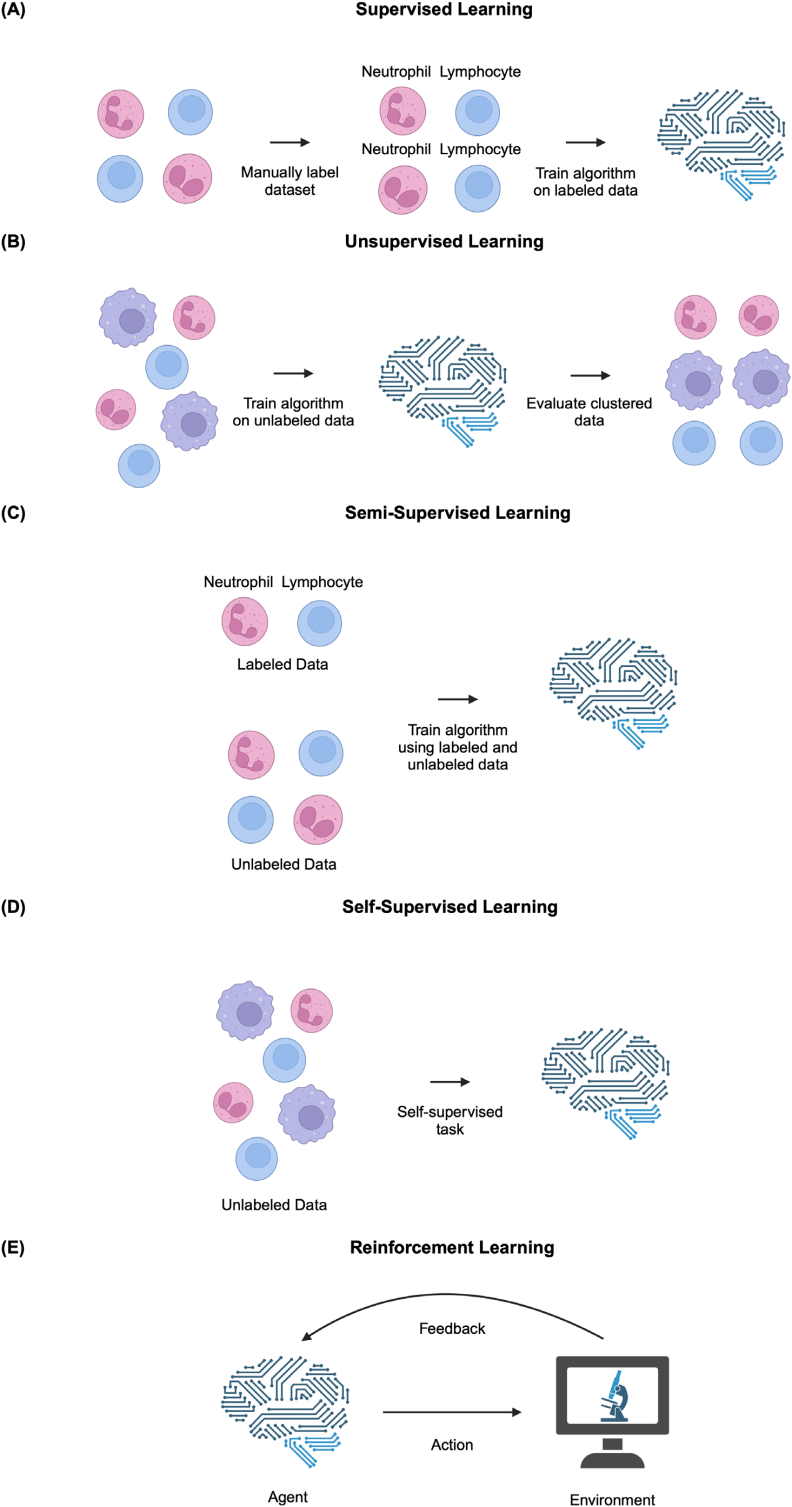
Visual representations of learning paradigms. (A) In supervised learning, a labeled data set is used to train (or fit) the algorithm, producing a model that is then evaluated on a test data set to evaluate performance. (B) In unsupervised learning, an algorithm is trained on an unlabeled data set. The algorithm groups or clusters similar data points, and the resulting clusters can be analyzed to identify associations within the data. (C) In semi‐supervised learning, an algorithm is trained using both labeled data and unlabeled data. The labeled data can be leveraged to guide the algorithm in making predictions about the unlabeled data, which can then be used as pseudo‐labels to further expand the labeled training set. (D) In self‐supervised learning, algorithms are trained on entirely unlabeled data by generating their own tasks, such as predicting missing parts of the data. This process allows the model to learn useful representations that can be applied to downstream tasks. (E) In reinforcement learning, an agent interacts with an environment by taking actions. The environment provides feedback in the form of rewards or penalties based on the actions taken. The agent learns over time by adjusting its actions to maximize cumulative rewards, resulting in improved performance in the task it is trained to complete. Created with BioRender.com.

Machine learning (ML) is a subtype of AI that underpins most modern AI‐based applications (Figure 1). In contrast to traditional programming, which requires explicit, hand‐crafted rule sets, ML algorithms learn patterns or representations directly from data [9, 10]. Consequently, ML methods are often categorized into paradigms by how they utilize data. Across all ML paradigms, the central principle is that algorithms iteratively adjust their internal parameters based on feedback from data, rather than following fixed rules manually encoded by a human programmer. Five widely recognized ML paradigms are supervised learning, unsupervised learning, semi‐supervised learning, self‐supervised learning, and reinforcement learning (Figure 2).

### 3.1 Supervised Model Development

**FIGURE 3:**
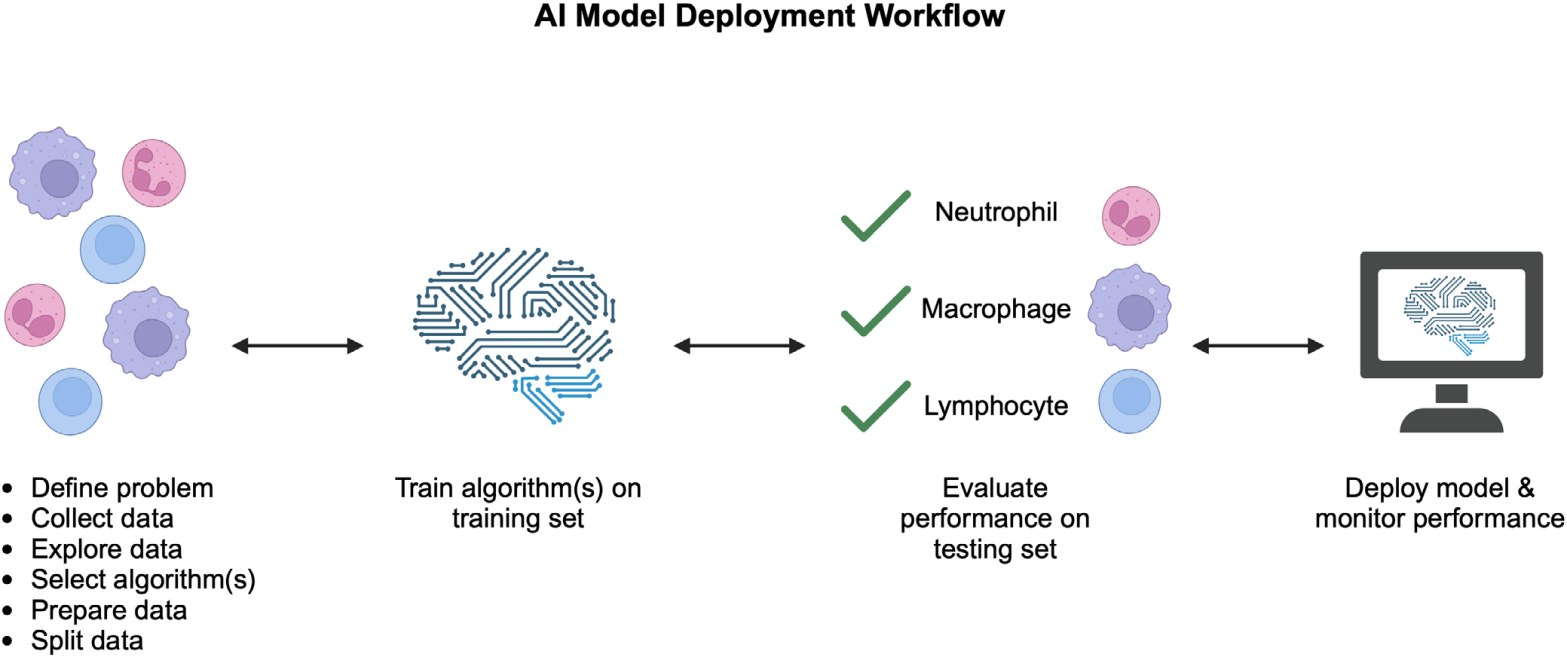
Visual representation of AI model deployment workflow. At the outset of ML model development, several steps are required, including (1) defining the problem to be addressed, (2) collection of necessary data, (3) exploration of the data, (4) selection of the initial algorithm(s) to be trained, (5) preparation of the data, and (6) splitting of the data into training and testing sets. The algorithm(s) is then trained using the prepared training set. Model performance is then evaluated using the prepared testing data set. If the model is performing acceptably, the model can be deployed and monitored to ensure continued high performance on real‐world data. If the initial testing produces suboptimal results, or performance drifts or decays following deployment, additional training and testing may be undertaken to return the model to rigorous performance. Created with BioRender.com.

Supervised learning uses data with labels, which are also called the dependent variable or target variable, specifying the correct output for a series of input variables, which are also called the independent variables or features (Figure 3) [9, 10]. Two tasks supervised learning can be used for are classification and regression. Classification results in a discrete label (e.g., “neutrophil”) being assigned to the input, whereas regression predicts a continuous value (e.g., 1.035) based on the input.

As an example of classification, consider an intended use whereby a clinical pathologist would like a classifier to differentiate between neutrophils and nonneutrophils and subclassify segmented and band neutrophils from a peripheral blood smear. Such a use case could be part of a wider set of AI models that collectively perform automated blood smear evaluation and provide preliminary results to the clinician while the sample awaits final clinical pathologist review. A set of images would first be labeled (annotated) by a trained expert to differentiate cells as either segmented neutrophil, band neutrophil, or not neutrophil. A supervised algorithm would be trained on the labeled images, resulting in a model, f, that takes an image as input, x, and classifies that image as either neutrophil (with subclass) or not neutrophil, *Y* (*Y* = *f*(*x*)).

Now, for an example of regression, consider instead an intended use whereby a clinical pathologist would like an AI model to evaluate CBC and chemistry profiles and propose a urine diagnostic endpoint such as specific gravity. This could help support a clinician in determining whether a cat who only had bloodwork done without a urinalysis needs to return for urine collection and evaluation. A set of variables from the CBC and chemistry profile (e.g., hematocrit, creatinine, blood urea nitrogen) of cats would be selected as the input variables, and the urine‐specific gravity of the cats could be used as the label (and thus the output variable). A supervised algorithm would be trained on the labeled bloodwork data, resulting in a model, f, that takes CBC and chemistry profile data as input, x, and outputs a predicted urine specific gravity, *Y* (*Y* = *f*(*x*)).

There are various AI algorithms that can be used for supervised learning. Common supervised AI algorithms include but are not limited to linear regression, polynomial regression, logistic regression, k‐nearest neighbors, support vector machines, naïve Bayes, decision trees, random forests, and neural networks (discussed later). Algorithm selection is typically based on the goal of the project (e.g., classification or regression), the complexity of the data (evaluated through exploration of the data), and an iterative process of training, testing, and comparing different models.

Data sourcing is integral to model development. A main consideration for data sourcing is the use case of the model. If the tool is to be utilized by a single institution, the data used for training and testing can likely be sourced solely from that institution. However, if generalizability is desired, it is important to incorporate data from a variety of sources that reflect the variability expected to be encountered by the model once in use (e.g., variability in laboratory methodologies and patient populations). Even subtle differences across data sets can degrade model performance if they are not accounted for during model development. Various strategies exist to mitigate any challenges that arise from sourcing data from multiple institutions. These include collaborative standardization of data collection and storage protocols across institutions, the creation of multi‐institutional central data repositories, and federated learning approaches, where AI models can be trained on multiple decentralized devices, negating the need to centralize or share data. Various data sets and data set repositories are also available publicly online, but care should be taken to ensure they contain quality data. There are efforts to create large, high‐quality pathology nonclinical and clinical image repositories, such as Bigpicture, but to the authors' knowledge, most efforts to date focus on human tissue histopathology [11].

After data acquisition, data exploration is conducted to inform subsequent steps. Data preparation (so‐called “cleaning”) is then typically required. Algorithms typically require input variables to be numerical values, so categorical values require conversion to a numerical representation. Care must also be taken to ensure numerical data is properly formatted, especially if data are sourced from multiple locations. For example, data being sourced from two laboratories that use different units of measurement should be converted.

Missing values are also frequently encountered even in highly curated data sets due to numerous reasons including but not limited to variability in reporting practices across institutions, sample availability, and incomplete records or clerical mistakes. Imputation is the process of filling in these missing values with substituted values. Various imputation approaches are taken depending on the situation, ranging from replacing missing values with a measure of central tendency (mean/median/mode) to creating another ML model to predict the missing value [10, 12]. Data are also often scaled and/or normalized to ensure that variations among different features do not skew model performance, preventing any single variable from disproportionately influencing the model.

Image data sets also require preprocessing, especially given the large file size of most pathology images. Common steps include color normalization to correct for staining variations, noise reduction to clarify structural details, and resolution adjustment for manageable image sizes. Techniques also exist to isolate regions of interest and remove artifacts [13, 14]. Given the potential of AI‐driven image analysis in veterinary clinical pathology, it is suggested that readers explore the numerous resources on the topic, especially those focused on veterinary histopathology [15, 16, 17].

Finally, data sets often suffer from imbalances and inherent biases that will result in imperfect model performance when applied to unseen data. To mitigate these issues, techniques such as oversampling and undersampling are employed. Oversampling involves artificially increasing the presence of underrepresented classes in the data set, whereas undersampling reduces the prevalence of overrepresented classes, thereby balancing the data set. For example, in a data set composed of bronchoalveolar lavage (BAL) cytology WSIs from healthy animals, where each cell is labeled, the prevalence of macrophages and the scarcity of eosinophils can skew an AI model's ability to accurately count cells in BAL samples from both healthy and sick patients. In such cases, undersampling the overrepresented macrophages and oversampling the underrepresented eosinophils can help create a more balanced data set, leading to more reliable AI predictions. Advanced strategies exist to handle imbalanced data sets but are beyond the scope of this review [18, 19].

After data exploration and preparation, the data set is typically split into training and testing sets. This is done so that models can be tested on data not used in training, ensuring that model performance metrics are not artificially inflated due to learned patterns specific to the training set. This split is often done by random sampling, with 80% of the data commonly allocated to the training set and 20% to the testing set. Alternative ratios may be utilized depending on the characteristics of the data set, and research has explored defining the ideal split [20, 21]. Popular machine learning libraries for Python, such as scikit‐learn, offer convenient methods for splitting data sets as needed [22]. Once the split is made, models are trained, or fit, using the training set, and tested on the testing set to evaluate model performance on novel or unseen data. In more complex models, a third set called the validation set is often introduced to further refine model performance.

However, relying on static data splits can be limiting, as it assumes the split is fully representative of the broader data set. Cross‐validation techniques are commonly employed to address this issue, especially in smaller data sets where variability can impact model performance. One such technique is K‐fold cross‐validation [9]. This consists of further splitting the training set into k number of folds or groups. The algorithm is then trained k times, leaving out a single different fold each time, and on each training iteration, model performance is assessed on the fold left out. This allows for the creation of a more generalizable model as it assesses model performance across the entire data set as opposed to a single subset.

ML algorithms also have hyperparameters, which are considered external to the algorithm as they must be set by the individual creating the model [9, 10]. Hyperparameters consist of configurable variables such as learning rates, the number of trees in a random forest, or the number of nodes in a neural network (discussed later). Hyperparameters are typically tuned or optimized during the iterative training process. Numerous methods exist for hyperparameter optimization [23, 24].

Model performance for classification tasks is evaluated using many metrics familiar to veterinary clinical pathologists, including accuracy, precision, sensitivity (often called recall in ML), specificity, and area under the curve (AUC) of the receiver operating characteristic (ROC) [10]. *F*_1_ scores, the harmonic mean of precision and sensitivity (recall), are frequently reported, as *F*_1_ scores allow for performance to be summarized by a single numerical value that considers the impact of both false positives and negatives [10]. *F*_1_ scores are particularly valuable in cases with imbalanced data sets—a common scenario in real‐world data—where models that perform well on overrepresented classes can still yield misleadingly high accuracies despite poor performance on underrepresented classes. Balanced accuracy is also frequently used in cases with imbalanced data sets and typically refers to the arithmetic mean of sensitivity and specificity in binary classifications and the average sensitivity across each class in multiclass classifications. Confusion matrices, similar to those clinical pathologists use to calculate sensitivity and specificity in classical diagnostic testing, are also frequently used to visualize model performance. There is an ongoing effort in image analysis to ensure proper usage and interpretation of metrics [25, 26].

Different metrics are required for the evaluation of regression tasks because the output variable is continuous rather than categorical. Common metrics include mean absolute error (MAE), mean squared error (MSE), root mean squared error (RMSE), R‐squared, and adjusted R‐squared.

Models are often said to be over‐ or underfitted when evaluating their performance [9, 10]. Overfitting refers to when a model learns the training data set too well, capturing noise and outliers, which harms model performance on unseen data. With overfitting, one expects to see a higher *F*_1_ score on the training data set than on the testing data set. Alternatively, underfitting refers to when a model is too simplistic and fails to capture the patterns in the training data, resulting in poor performance metrics in both the training and testing set.

### 3.2 Other Learning Paradigms

Unsupervised learning models, as the name suggests, identify patterns in data without requiring explicit labeling of the desired outputs from the input data [9, 10, 27]. In unsupervised learning, data are not labeled, so performance metrics like those discussed above cannot be calculated, making it challenging to assess the performance of these models. Typically, unsupervised learning is tasked with exploratory data analysis. Two such applications are dimensionality reduction and clustering.

Dimensionality reduction decreases the dimensions of larger data sets to enhance interpretability while minimizing information loss. Principal component analysis (PCA) is a common method used for dimensionality reduction. Simplistically, PCA takes a larger set of correlated variables and reduces this into a smaller number of variables that still collectively explain most of the variability in the nonreduced data set. PCA broadly allows for easier visualization of multidimensional data sets and identification of key relationships within the data. For example, consider a data set consisting of EHRs of dogs with a chronic disease with an unknown etiology. Using PCA, endpoints contributing the most to the variability in the data sets can be identified, perhaps leading to the identification of novel associations and the underlying unknown etiology of the chronic disease.

Clustering refers to numerous techniques for finding subgroups within data. This can uncover previously unidentified relationships, such as subgroups within a disease. For example, consider again the group of dogs with the chronic disease whose etiology is now known. Clustering could be used on the EHR data set to uncover subgroups within the disease, perhaps uncovering breed‐specific susceptibilities or responses to treatment.

Semi‐supervised learning bridges the gap between supervised and unsupervised learning, ideally suited for scenarios with partially labeled data sets [9, 10]. This methodology leverages various strategies to use labeled and unlabeled data effectively. Typically, the labeled portion of the data set is used to make informed predictions about the unlabeled data, thereby augmenting the training set and enhancing the model. This approach is increasingly pertinent in the era of large‐scale data. Let us suppose a data set consists of 10 000 cytology WSIs, some of which are labeled with various diagnoses. The labeled WSIs could be used by a semi‐supervised AI model to make predictions about the unlabeled images, enriching the data set and improving the training of the AI model without requiring many hours of labeling by an expert cytopathologist.

Self‐supervised learning is where models learn to generate their own labels from the data itself. Models create tasks, such as predicting missing words in a sentence or reconstructing an image from a distorted version, that allow them to learn meaningful patterns and representations without needing explicit labels. Self‐supervised learning is frequently used in the generation of pretrained models, which can then be fine‐tuned for specific tasks with minimal labeled data. This approach underlies many of the generative AI tools that are currently capturing global attention (discussed later).

Reinforcement learning is where an agent learns to make decisions by taking actions in an environment to achieve some goal [9, 10]. The agent learns from the consequences of its actions, rather than from being told explicitly what to do. At each step, the agent receives feedback in the form of rewards or penalties, guiding it to adjust its strategy to maximize cumulative rewards over time. Reinforcement learning is most frequently utilized in complex tasks in the physical world, such as autonomous driving or robotics.

As a relevant example, a busy diagnostic laboratory that wishes to prioritize samples based on clinical utility (measured simplistically through clinician contact for the sake of the example; in reality, other factors would have to be considered, such as patient stability and case complexity) as opposed to first in, first out. A reinforcement learning agent is tasked with this optimization of sample prioritization. The agent interacts with a virtual environment representing the clinical pathologists' worklist. The agent selects an action that corresponds to placing a sample in a particular spot in the ordered worklist. The agent receives positive feedback from a clinician expressing appreciation for the quick result or no clinician contact. The agent receives negative feedback for a clinician contacting the clinical pathologist for a preliminary result prior to report finalization if enough time elapses between sample receipt and when the pathologist would have realistically been able to finalize the report if the sample had received a higher priority ranking from the agent. Over time, the agent learns from the consequences of its actions, adjusting its prioritization strategy to maximize positive feedback and, thus, sample prioritization.

### 3.3 Neural Networks

**FIGURE 4:**
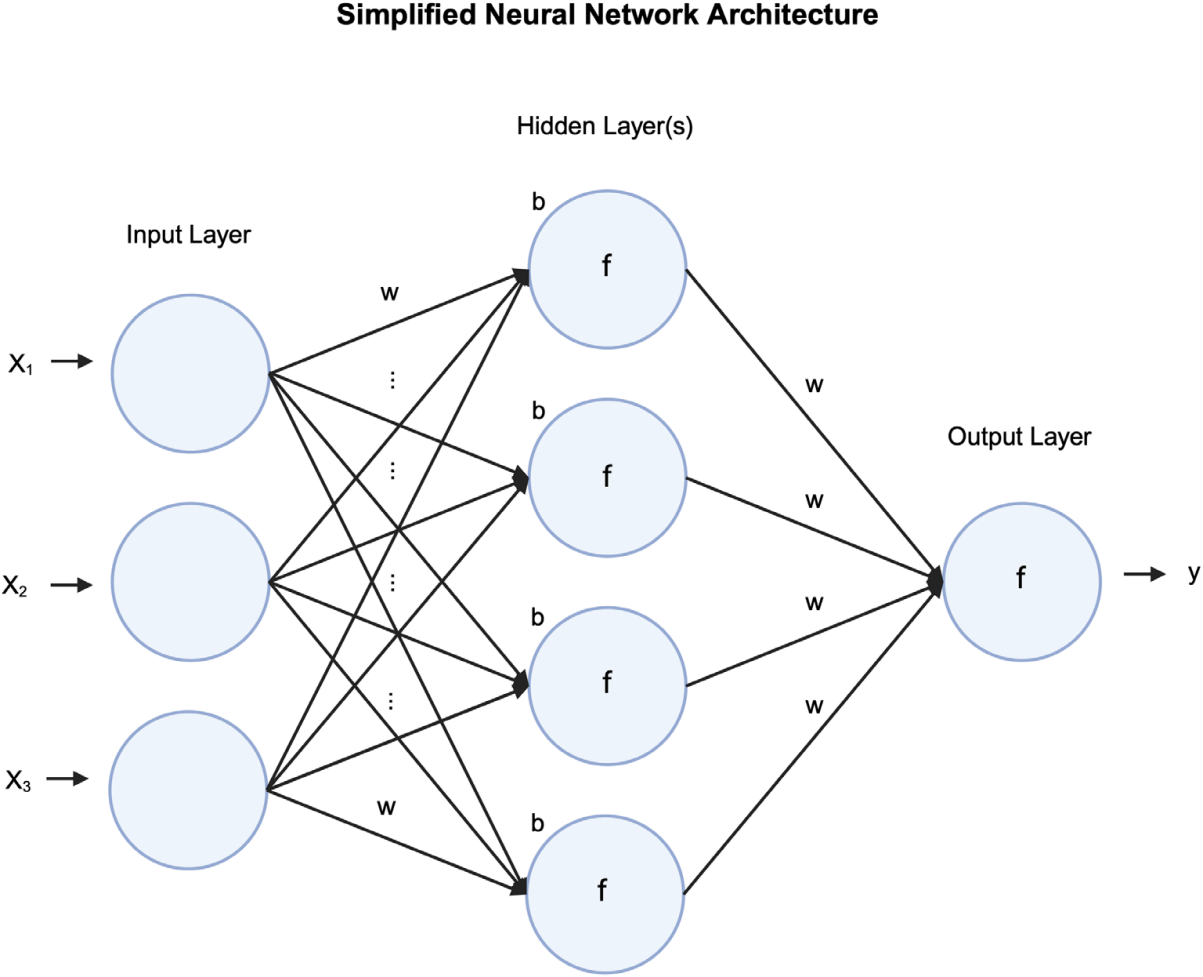
Simplified neural network architecture. The first layer of a neural network is the input layer. Each node in the input layer corresponds to one independent variable from the input data (three in this example). These features are then passed to the hidden layer(s). There can be one hidden layer (shallow neural network) or multiple hidden layers (deep neural network). Each connection between nodes has its own weight (w), each node has its own bias term (b), and each node has its own activation function (f; e.g., Rectified Linear Unit or ReLU). At each node, the input is multiplied by its corresponding weight, the weighted inputs are then summed, the node's bias is added to the summation, and the summation is passed through the activation function (*f*(∑(*w*_*i*_·*x*_*i*_) + *b*)). The result is then passed to the next hidden layer or the output layer. The output layer transforms the incoming features from the final hidden layer into a format suitable for interpretation as a solution to the problem being addressed (y; e.g., single value for regression tasks, probability distribution across several classes for classification tasks). The output layer can consist of a single node (for binary classification or a single regression task) or multiple nodes (for multiclass classification or multiple regression tasks). The activation functions in output layers are crucial as they shape the format of the output (e.g., sigmoid, softmax, linear). The number of hidden layers, the number of nodes in each layer, and activation functions are hyperparameters set before model development, while weights and biases are set autonomously by the model during training. Created with BioRender.com.

Artificial neural networks (ANNs) are a class of ML algorithms inspired by biological neural networks and were built to solve complex problems by mimicking the way biological neurons signal one another (Figure 4) [9, 10]. In ANNs, nodes, or neurons, receive the input, process it, and pass it on to the next layer of nodes. Nodes are organized into layers, including an input layer that receives the data, single or multiple hidden layers that perform computations, and an output layer that delivers the result. Each connection between nodes has an associated weight and each node may have a bias, both of which are adjustable parameters that are potentially changed during model training. Nodes can also have an activation function, which allows for increased complexity.

During training of a neural network, input data are fed through the network, called forward propagation, until it reaches the output layer, producing a prediction. The loss function then measures the error between this prediction and the actual value, and this error is propagated back through the network, called backpropagation, to adjust the weights and biases. Using an optimization technique such as gradient descent, the weights and biases are adjusted to minimize the error and improve the model.

ANNs can be shallow, containing a single hidden layer between the input and output layer, or deep neural networks (DNNs), which contain more than one hidden layer. DNNs are the basis of deep learning (DL), which is largely driving the frequent AI advancements seen today [9, 10]. While the numerous hidden layers in DNNs drive their success, they also lead to difficulty in understanding how DNNs reach a decision, resulting in their decision‐making process being termed a “black box.” This lack of transparency emphasizes the need for supervision of the models by an expert (e.g., clinical pathologist) and the ability to reject an output and add new input (training) followed by testing and validation of model versions. The concept of a “black box” can be worrisome when complex decisions are reached, especially in sensitive areas like health care. Explainable AI (XAI) has attempted to resolve this through various methods beyond the scope of this review [9, 10].

Convolutional neural networks (CNNs) are a subset of DNNs designed for handling data in a grid‐like topology, such as images, and have transformed the field of computer vision (domain centered on enabling computers to interpret visual information), with countless successful applications across image‐based scientific disciplines like radiology and pathology [9, 10]. A key aspect of CNNs is convolutional layers, which slide filters over the data (e.g., image), capturing features such as edges, textures, or other patterns.

Recurrent neural networks (RNNs) are another subset of DNNs designed for handling sequential data, such as in natural language processing and time series prediction [9, 10]. RNNs have a memory capacity of sorts, though limited, as each element in the data set is passed along with context considered from previous elements.

Transformers, another type of DNN that was first introduced in 2017 and has revolutionized modern AI, process entire sequences of data in parallel instead of sequentially like RNNs [9, 10, 28]. Simplistically, this is achieved through a novel self‐attention mechanism, allowing the model to capture context throughout the sequence and thus model relationships between input data. These models are often pretrained on vast amounts of general data (so‐called “foundation models”) and then fine‐tuned on smaller, task‐specific data sets (see previous discussion of self‐supervised learning). Transformers underlie the large language model (LLM) chatbots and generative AI tools currently capturing global attention, such as ChatGPT, Claude, and Gemini, as well as image and video generative models such as DALL‐E and Sora [29]. These multimodal models, meaning they take in multiple types of data such as text and images, take input and respond by generating text, images, or videos that are contextually relevant. While their responses seem as though they are surely written by a human, they are generated based on statistical patterns learned from extensive data analysis.

LLMs notoriously suffer from hallucinations, where models refer to incorrect, fabricated, or nonsensical information that is not supported by the input data or training data [30]. This is particularly concerning in medical fields, where it could lead to harmful consequences. For example, an LLM tasked with the generation of cytopathology reports may confidently diagnose an entirely fictitious entity or, perhaps more concerning, confidently misdiagnose the sample with a real but incorrect entity. Numerous approaches have been created to help address hallucinations, with one promising approach being the inclusion of source citations in model outputs. Another promising approach is the utilization of retrieval‐augmented generation (RAG) [31]. Simplistically, RAG utilizes a retrieval system and a curated database alongside a generative AI model to help ground responses in contextually relevant material. Simply improving the training set of models also reduces hallucinations. Overall, the possibility of hallucinations further highlights the need for veterinary clinical pathologist oversight of any AI model implementation.

Vision transformers (ViTs) are algorithms that apply the transformer architecture to computer vision tasks [32]. Given transformers' unique ability to capture context, ViTs potentially capture global interactions that CNNs cannot. However, ViTs also require larger amounts of data than CNNs, which are frequently not available in fields like veterinary pathology.

## 4 Qualification and Validation of AI Applications

One of the most important responsibilities of the veterinary pathologist is ensuring our methods are scientifically valid and reproducible. New technology, if not applied systematically with the correct controls and documentation in place, can be detrimental to our mission of serving our various stakeholders, including veterinarians and physicians, regulators, and veterinary or human patients. While qualification and validation of a new method can appear onerous, our field has a long history of doing this across laboratories supporting veterinary diagnostics and research and development [33].

Stakeholders do vary across industries and influence the approach to qualification and validation. In human pathology practice in the United States, a combination of federal standards for diagnostic (Clinical Laboratory Improvement Amendments or CLIA) or clinical‐based research (Good Clinical Practices or GCP) laboratories is under the oversight of the FDA [34]. If the methodology is to be marketed for human clinical diagnostic use as a medical device, the extent of device evaluation is dependent on how severe the impact a device malfunction would have on the intended patient population (Class I low, Class II medium, Class III high). To date, digital pathology (scanners, viewers, software) and AI‐based tools marketed for use in medical pathology, like Phillips Intellisite, have been considered Class II devices (medium risk) by the FDA and follow a 510(k) premarket clearance or notification [35]. In this process, the manufacturer provides supportive safety data and notifies the FDA of their intent to market a medical device (the Premarket Notification or PMN). Regulators then determine whether the device is equivalent to a device already placed into one of the three classification categories (Class I, II or III).

For nonclinical digital pathology and AI applications used in support of research and development, validation guidelines are also provided by government regulators. In the United States, the FDA's Code of Federal Regulations (21 CFR Part 58) defines Good Laboratory Practices (GLP) [36]. GLP is a framework for conducting well‐controlled nonclinical studies and is a benchmark globally for nonclinical research excellence. Within the 21 CFR, there are also regulations for computer system validation (Part 58) and the use and management of electronic records (Part 11), both important for digital pathology and AI methods implementation [37]. Parts 11 and 58 do not apply to the development of digital pathology and AI‐based methods for veterinary diagnostics. However, the principles behind these regulations drive data quality and integrity as well as support the reconstruction and repeatability of research and are an important part of any veterinary diagnostic method development. Many companies and industries have adopted Good Research Practices (GRP) that support these widely adopted scientific practices in research [38]. The FDA, Health Canada, and the United Kingdom's Medicines and Healthcare Products Regulatory Agency also jointly developed guiding principles for Good Machine Learning Practice (GMLP) for medical device development, which provide a framework for the safe and ethical development of ML models in health care [39].

**FIGURE 5:**
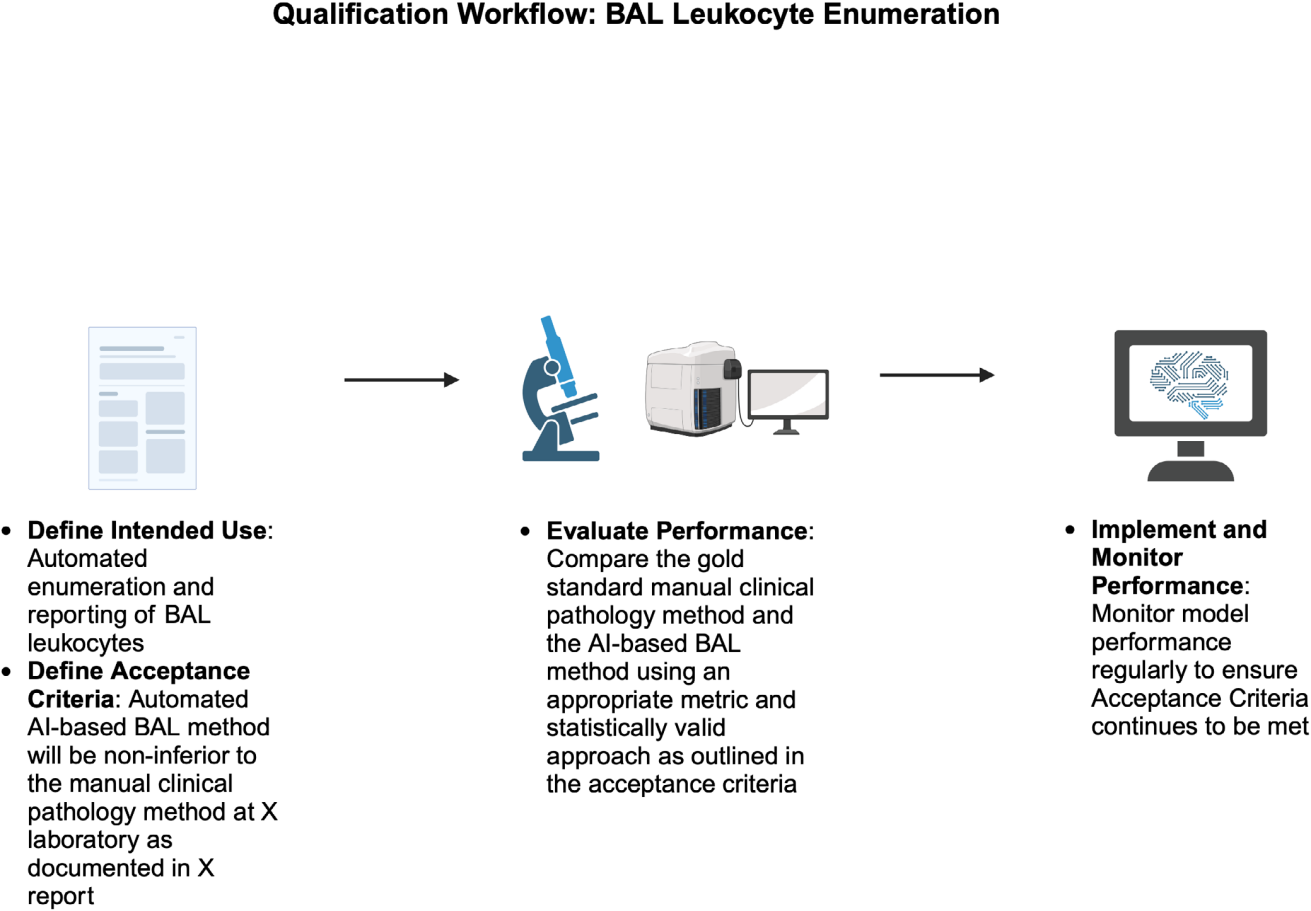
AI method qualification workflow: BAL leukocyte enumeration. Qualification is the process of demonstrating whether a method (in this case, an AI‐based method for enumerating and reporting BAL leukocytes) satisfies its intended use. The workflow begins with clearly defining the intended use of the AI tool. Following this, the acceptance criteria, or gold standard, must be defined for the method using an industry benchmark, relevant literature, or internal data. An example of acceptance criteria could be that the method of interest is noninferior to the identified gold standard, determined by comparing the two approaches using relevant statistical analyses. Once the qualification plan is clearly defined, the AI method's performance can be evaluated, and if the method meets the acceptance criteria, the method can be implemented. Performance should be continuously monitored even after qualification. Created with BioRender.com.

For the purposes of this review, we will consider qualification as the evaluation of a method resulting in the demonstration (or not) that the method will satisfy the intended use as defined by the veterinary pathologist (Figure 5). Qualification is a component of the broader concept of validation, which includes the evaluation of the new method itself and the process and documentation underlying the generation of the data, its evaluation, and reporting. Qualification (and validation) plans require a clear definition of the intended use. For example, the qualification plan for an AI‐based method to provide the automated enumeration and reporting of BAL leukocytes will be different from a method that provides an alert to a clinical pathologist for a secondary review of a BAL sample that demonstrates a variance of X% total cellularity from a historical control range in a veterinary patient population.

After the intended use is defined, the veterinary pathologist needs to define acceptance criteria (“the gold standard”) for the method. If one uses the automated BAL enumeration intended use as an example, the clinical pathologist should determine the gold standard from an industry benchmark, the literature, or internal data. In the BAL example, if internal data is used, the clinical pathologist could design a study that determines the sensitivity and specificity of the human evaluation of BALs within their laboratory. Then using these data, the clinical pathologist would define the acceptance criteria for the AI method. An example could be “the automated AI‐based BAL method will be non‐inferior to the manual clinical pathology method at X laboratory as documented in X report.” Noninferiority will be characterized by some type of appropriate metric(s) comparing the gold standard and the AI method using a statistically valid approach, as described earlier in this review. The statistical measures used will depend on what is compared, like cell type(s) identification, diagnosis, or a value or combination of continuous or non‐continuous data (severity scores). AI applications in medical practice (veterinary and human) will continue to evolve, as will newer methods to support their qualification and ensure the best performance possible [40]. Quality assessment standards and monitoring will be important and dependent on the veterinary clinical pathologist's oversight with support from biostatisticians, technical specialists, and quality control scientists [41].

Later in the manuscript, specific examples of AI applications for Veterinary Clinical Pathology are provided. One is Zoetis Diagnostics' Vetscan Imagyst (which utilizes the Grundium Ocus 40 slide scanner and Techcyte's AI technology), an AI deep learning‐based tool that has the intended use for supporting veterinary practitioners in the diagnostics of urine sediment preparations, blood smears, dermatologic samples (skin impression smears, ear swabs, and skin swabs), and fecal samples [42]. In two manuscripts, Zoetis scientists describe a qualification methodology for the fecal sample AI evaluation method in dogs and cats that reflects several common themes [43, 44]. First, since there was no industry benchmark for fecal sample performance metrics (sensitivity and specificity), consistency in analytical methodology (passive or centrifugal techniques), or staff completing the procedure (parasitologist, veterinarian, or technician), Zoetis completed controlled studies that compared both manual technical methods (passive and centrifugation) with the AI workflow output. The diagnostic data for both methods were evaluated using standard two‐by‐two (2 × 2) tables and calculations of sensitivity and specificity. They also examined the performance of the flotation methods as well as time and motion data for the different workflows (manual vs. AI). These latter studies are important for guiding real‐world applications of the method in veterinary practice.

For both slide preparation methods, the Vetscan Imagyst diagnostic result, compared with the parasitologist, had a high Pearson's correlation coefficient (*r*), which was considered noninferior to the manual practice. The studies also documented some limitations to the AI workflow. For example, in the dog study, Vetscan Imagyst could miss eggs at the edge of the coverslip that a parasitologist would identify [43]. This limitation was noted when a larger amount of the sample was placed on a fecal slide, which is important information that would support standard operating procedures for a veterinary laboratory (define volumes that should be used and a secondary manual examination if volumes are above specifications). Finally, the authors note that because the AI method is deep learning‐based, it can be constantly improved using methods discussed previously in this manuscript. How improvements are made and documented, and the process of releasing new deep learning‐based classifiers should be controlled by procedural methods in the laboratory.

A detailed discussion of regulatory guidelines (21 CFR Part 11 and 58) in reference to GLP validation of an AI‐based method is beyond the scope of this review and not applicable to veterinary diagnostics [36, 37]. However, the application of principles from these guidelines is important in the delivery of an AI‐based method. First, standard documentation of procedures with the appropriate controls (standard operating procedures) and job descriptions, as well as training of staff in place, is necessary for support of any laboratory method. Also important is a chain of custody for the sample and data (from sample generation to diagnostic report) and an audit trail (for changes in the data supporting the diagnostic decision like retesting). Second, the industry benchmarks associated with computer systems validation (see What Is Computer System Validation?) and information security (see ISO 27001) are useful reference guidelines for a software‐based solution such as an AI‐based method [45, 46]. Finally, quality systems management approaches ensure procedures are followed (quality reviews), issues or unplanned events are tracked (deviations), and solutions are documented (corrective and preventive actions [CAPAs]). There is a quality management systems guideline (see ISO 13485:2016) for human medical devices that represents a best practice for the development of AI‐based methods in a veterinary diagnostic laboratory [47].

## 5 AI Applications Relevant to Veterinary Clinical Pathology

This section reviews current use and research into the use of AI in domains relevant to veterinary clinical pathology. Veterinary‐specific applications are emphasized, but the number of existing applications not explored in the veterinary literature necessitates the inclusion of relevant human literature. However, it is important to note that AI applications in human pathology often do not directly translate to veterinary pathology for various reasons, including differences in the availability of large data sets, established grading schemes, and universal gold standards, as well as variable availability and statistical validation of prognostic data. Additionally, many of the studies discussed below are single institutional studies with relatively small sample sizes. These represent important proof of concepts but likely require significant expansion to generalize well.

### 5.1 AI‐Augmented Point‐of‐Care Testing

AI augmentation can enhance point‐of‐care (POC) instruments, allowing for closer to diagnostic laboratory performance in clinic. Several such diagnostic tools are currently in use in veterinary medicine. As described earlier, Zoetis Diagnostics' Vetscan Imagyst is an in‐clinic diagnostic tool that allows for AI‐driven analysis of urine sediment preparations, blood smears, dermatologic samples (skin impression smears, ear swabs, and skin swabs), and fecal samples [42]. It also allows for the scanning and transfer of digital cytology images for review by board‐certified veterinary clinical pathologists. IDEXX SediVue Dx Urine Sediment Analyzer utilizes AI to provide POC urine sediment analysis [48]. It performs well across a variety of tasks, although studies suggest manual review should be performed concurrently to confirm results, especially regarding the presence of epithelial cells, bacteria, and crystals [49, 50, 51]. In human medicine, HemoScreen and Hilab reportedly replicate laboratory‐quality CBC‐equivalent results using AI [52, 53]. Additionally, Demir et al. developed an AI‐based method to diagnose urothelial carcinoma in humans with high sensitivity and specificity using image analysis of droplets of blood and urine [54].

### 5.2 Multimodal Clinicopathologic Modeling

Multimodal AI‐based approaches that incorporate a wide array of clinical, clinicopathologic, and other relevant diagnostic data show potential to enhance diagnostic and prognostic capabilities. Such approaches show promise in improving the diagnosis of historically difficult‐to‐diagnose diseases. Dunbar et al. utilized signalment and laboratory data in an attempt to improve feline infectious peritonitis (FIP) diagnosis, resulting in a sensitivity of 95.45% and a specificity of 98.28% when evaluated on cases diagnosed with FIP via histology, immunohistochemistry, and/or PCR or an alternative non‐FIP diagnosis [55].

Other approaches have attempted to utilize laboratory‐generated data in conjunction with other diagnostic information as an alternative to invasive techniques (e.g., biopsy). Awaysheh et al. utilized ML models to differentiate between inflammatory bowel disease, alimentary lymphoma, and lack of disease in cats with an average sensitivity of 70.8% across 10 repeats of 10‐fold cross‐validation using CBC and serum chemistry values (average specificity was not reported across the 10 repeats but was reported for a single run with an average specificity of 85.4% across the different disease states) [56]. Similarly, Basran et al. attempted to combine small intestine ultrasound image data, CBC data, and serum chemistry data to differentiate between lymphoma, inflammatory bowel disease, a lack of disease, and other conditions in cats [57]. This failed to produce accurate models differentiating these states but resulted in high‐performing models for determining if a cat would benefit from a biopsy or not, reporting an accuracy of approximately 95%, which has clear implications for reducing unnecessary invasive testing.

Multimodal ML models using laboratory data have also shown prognostic capabilities in veterinary medicine. Kim et al. trained an ML model to use CBC and serum chemistry data alongside other clinical data, such as signalment, echocardiographic measurements, and blood pressure, to predict the risk of heart failure in dogs with myxomatous mitral valve disease with a sensitivity of 85.5%, a specificity of 68.9%, and an AUC of 0.88 [58]. Renard et al. created ML models to predict survival using laboratory data with other clinical data in cats with acute on chronic kidney disease (CKD) which generally showed high sensitivity and specificity for predicting survival at 7, 30, 90, and 180‐day time points [59]. Bradley et al. utilized serum chemistry and urinalysis data with age to create a model capable of predicting CKD in cats near the point of diagnosis with a sensitivity of 90.7% and a specificity of 98.9% [60]. Model performance degraded further out from diagnosis but still achieved sensitivities of 63% and 44.2% 1 and 2 years before diagnosis while maintaining a specificity of over 99%. Similarly, Biourge et al. created a model using serum creatinine and blood urea nitrogen with urine specific gravity to predict the risk in cats of developing CKD within 12 months with a sensitivity of 87% and a specificity of 70% [61]. Kokkinos et al. developed a model using creatinine, blood urea nitrogen, urine specific gravity, urine protein, weight, and age that could identify CKD in dogs with a sensitivity of 91.4% and a specificity of 97.2% [62]. The model also predicted the risk of developing CKD with a sensitivity of 68.8% at 1 year and 44.8% at 2 years.

Another use case for such multimodal models using laboratory data is screening for diseases with a relatively low index of suspicion. For example, Reagan et al. created a model that diagnoses hypoadrenocorticism in dogs with a sensitivity of 96.3%, a specificity of 97.2%, and an AUC of 0.994, using only CBC and serum chemistry data [63]. This model was then applied to a wide range of dogs in a clinical setting and showed a positive percent agreement of 100% and a negative percent agreement greater than 99% [64]. Similarly, ML models were created to diagnose hyperadrenocorticism, with the best‐performing model achieving a sensitivity of 71%, a specificity of 82%, and an AUC of 0.85 [65]. Reagan et al. also created an ML model using patient signalment, CBC, serum chemistry, and urinalysis data to predict leptospirosis in dogs with 100% sensitivity and 90.9% specificity, outperforming traditional acute serologic screening [66]. Similarly, Pijnacker et al. created an ML model that utilizes only ADVIA hematology data for prediction of *Babesia* parasitemia in dogs with a sensitivity of 100% and a specificity of 95.7% [67]. Torrecilha et al. were able to train an ML model to use serum chemistry values, physical signs, and serological testing to predict Leishmania infantum parasite load, typically quantified with qPCR in lymph nodes, with an accuracy of 0.869 [68].

Similar predictive applications in humans are numerous and can serve as inspiration for future implementations in veterinary medicine. One such focus with many publications is on the diagnosis, prognosis, and risk of sepsis in humans [69]. Kiss et al. also created an ML model for early prediction of acute necrotizing pancreatitis in humans using CBC and serum chemistry data with an AUC of 0.757 [70]. More generally, Rajkomar et al. used EHRs, including laboratory data, to predict a variety of important prognostic information, including in‐hospital mortality and prolonged stays, with an AUC of 0.93–0.94 and 0.85–0.86, respectively [71]. Yamao et al. utilized clinical data and laboratory data to predict the development of oliguria in human intensive care unit patients with AUC values of 0.964 at 6 h and 0.916 at 72 h [72]. ML models have also been used to predict mortality in human pneumonia patients, significantly outperforming classic scoring systems [73]. Other ML models predict the risk of mortality in COVID‐19 patients, disseminated intravascular coagulation, and pulmonary embolism [74, 75, 76]. Such applications continue to incorporate more diverse and expansive data sets, ranging from laboratory data to medical imaging data to genetic information.

### 5.3 AI‐Driven Image Analysis

There are relatively few examples of AI‐based image analysis in veterinary clinical pathology, though there are prominent examples in veterinary hematology and urinalysis. Several examples are discussed above in the AI‐Augmented POC Testing section [42, 48]. CellaVision is another widespread application that utilizes AI to expedite differential cell counts on blood smears, and Moichor is a commercial diagnostic laboratory that uses ML to perform automated avian and reptilian WBC differential counts and morphologic assessments as well as to enhance mammalian CBCs [77, 78, 79].

Despite this adoption in veterinary hematology and urinalysis, AI‐driven image analysis implementation has been relatively slow in veterinary cytopathology, especially compared to other imaging‐heavy disciplines such as radiology and histopathology. Given the success of such AI‐based image analysis in these other applications, it behooves veterinary clinical pathology to aggressively pursue such methodologies. This is further evidenced by the high performance of AI‐based approaches in the few examples in the veterinary literature. For example, Chu et al. developed an AI model to identify small, intermediate, and large lymphocytes in photomicrographs of canine lymph node aspirates, which was then used to diagnose lymphoma with an accuracy of 97.14% [80]. Marzahl et al. created a deep‐learning model that enumerates hemosiderophages in equine bronchoalveolar lavage fluid cytology samples with an average mean precision of 0.66 over five classes labeled on the slides and a concordance of 0.85, higher than the mean concordance observed between expert cytologists of 0.73 [81]. Interestingly, this model was also used to label additional equine, feline, and human bronchoalveolar lavage samples to create an accurately labeled database of hemosiderophages for future image analysis projects [82]. Further, Bertram et al. showed that ML algorithms improve reproducibility in the determination of total hemosiderin scores in equine bronchoalveolar lavage samples [83]. Sano et al. created an ML model for estrous cycle classification in mice that achieved an AUC of 0.962 and was similar in accuracy to human examiners [84]. While not cytopathology but relevant to veterinary clinical pathology, Smith et al. created a DL model that can perform bone marrow counts in Cynomolgus macaques on histologic bone marrow specimens with an 80.54% agreement between pathologists and the model [85].

The American Society of Cytopathology Digital Cytology Task Force recently released a two‐part paper with thorough considerations of the challenges and promises in digital cytology, including a thorough review of current AI applications available in human cytology practices [3, 86]. The use of AI in human cytology has a relatively long history centered around cervical cancer screening but also includes numerous other sites [87, 88].

Human studies have evaluated implementing AI into the interpretation of flow cytometry results with generally “expert level” accuracy, ranging from diagnosing Hodgkin's lymphoma, differentiating between various acute leukemias and myelodysplastic syndrome, subclassifying B‐cell non‐Hodgkin lymphomas, differentiating plasma cell myeloma from monoclonal gammopathy of undetermined significance, and automating the classification of normal cases [89]. There have also been numerous studies regarding implementing AI‐based models in serum protein electrophoresis evaluation in human medicine, with recent models achieving high performance [90].

### 5.4 AI‐Augmented Quality Control and Transformers in Pathology

Several human studies have also looked at using AI to augment laboratory quality control, recognizing sample mix‐ups and clotted samples as well as nondiagnostic cytology samples with promising results [91, 92, 93]. Further, given the revolutionary nature of LLM chatbots assisting professionals across numerous fields, the potential of LLMs has been explored in veterinary medicine broadly, and several human studies have assessed the ability of such models to assist laboratory professionals with promising but mixed results [94, 95, 96]. Finally, several human histopathology‐specific foundation models have been created recently [97, 98, 99]. These pathology‐specific models show great performance across a wide array of histopathologic assessments. The utility of such approaches is evidenced through PathChat 2, which is a multimodal generative AI copilot built using a pathology foundation model and an LLM that shows great promise as a general human pathology tool [100, 101]. Such an approach using cytologic and other clinical pathology images may allow for rapid and robust advancements in veterinary clinical pathology image analysis.

## 6 Conclusions

AI is a complex technology with great potential in the data‐rich field of veterinary clinical pathology. Many opportunities exist for its implementation across the entire field, ranging from quality control to diagnostic methodologies and data analysis. Certainly, challenges to AI implementation do exist. These include a lack of centralized databases, the need for improved slide scanning capabilities, and the variation across veterinary medicine (e.g., species differences, geographical variation, interlaboratory variations). However, the potential applications of AI in veterinary clinical pathology are clear, and as technologies continue to improve and new approaches are developed, these challenges will diminish.

It is also crucial to emphasize that AI models should be designed to augment, not replace, veterinary clinical pathologists. The active involvement of pathologists is essential throughout the entire AI lifecycle—from development to deployment and beyond. For example, once models are deployed, they must be monitored for degradation in performance due to variations in the environment, termed model drift. Further, current AI models can also only identify what they are trained to identify, meaning rare or novel diseases will be missed.

Ideally, veterinary pathologists will work together with AI‐interested and experienced colleagues to thoughtfully design, train, test, and implement responsible AI‐driven technologies that enhance and improve our efficiency and quality. Pathologists have important roles in clinical diagnostics and on translational research teams and possess critical expertise needed to act as the gold standard and vetting mechanism for ML‐derived information. Veterinary pathologists, our clinician colleagues, patients, and clients stand to benefit from the vast potential advancements to be made when pathologists and AI work in tandem.

## Conflicts of Interest

The authors declare no conflicts of interest.
